# Supplementary figures and images for: RBM15 Promates the Proliferation, Migration and Invasion of Pancreatic Cancer Cell Lines
Source: Cancers (Basel). 2023 Feb 8;15(4):1084. doi: 10.3390/cancers15041084 (PMC9954619; doi:10.3390/cancers15041084)

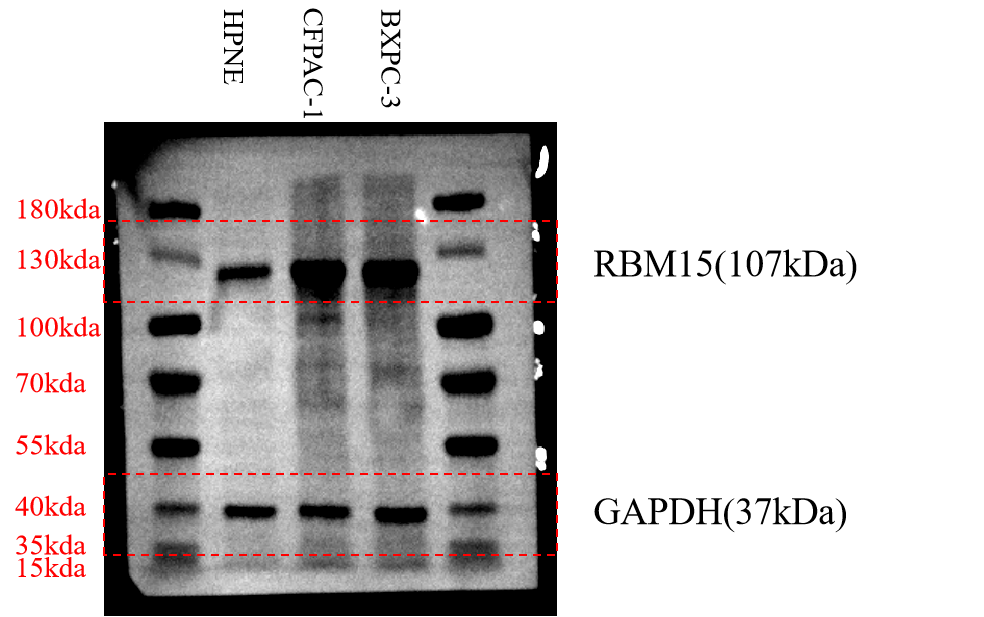

Supplement: Supplementary file 1 [file cancers-15-01084-s001.zip › cancers-2170069-Supplementary File S1.tif]
